# Virulent Purulent Sepsis

**Published:** 1887-06

**Authors:** 


### *VIRULENT PURULENT SEPSIS.*

At a late meeting of the Philadelphia Obstetric Society, Dr. Hirst presented specimens by permission of Prof. Parvin, from whose patient they were taken: "The specimens are interesting, not merely because they come from a case of puerperal fever, which unfortunately is not a rare disease, but from the rapidity with which the disease terminated fatally, and from the possible point of entrance of the septicæmic poison. The history of the case before delivery presents nothing worthy of note. Immediately after delivery the temperature was 99.5°, and in spite of the most energetic antiseptic treatment of the vagina and uterine cavity the temperature rose to 102°, but dropped again to 99.5°, only to rise again to 102°, where it remained until the woman's death about seventy two hours after the birth. The post-mortem examination showed diphtheritic patches in the vagina, extending into the cervical canal. The uterine cavity and walls were normal; the peritoneum tubes and ovaries healthy; the kidneys were the seat of numerous metastatic abscesses and there were several infarcts in the liver. The lungs were healthy. The rectum was covered with extensive patches of diphtheritic membrane; a very interesting condition, for it indicates the possibility at least that here was the point of infection, and if this is the case, this specimen at once assumes considerable importance, for he knows only three such cases in medical literature, one by Winckel, the others by Koster and v. Recklinghausen. These specimens may well serve to call attention to the possibility of infection by the administration of enemata and to the importance of observing the most minute precautions as to the chemical cleanliness of every instrument that may come in contact with the parturient or puerperal woman."

